# Host Biomarkers Reflect Prognosis in Patients Presenting With Moderate Coronavirus Disease 2019: A Prospective Cohort Study

**DOI:** 10.1093/ofid/ofac526

**Published:** 2022-10-06

**Authors:** Arjun Chandna, Raman Mahajan, Priyanka Gautam, Lazaro Mwandigha, for the PRIORITISE Study Investigators, Pragya Kumar, George M Varghese, Constantinos Koshiaris, Yoel Lubell, Sakib Burza

**Affiliations:** Cambodia Oxford Medical Research Unit, Angkor Hospital for Children, Siem Reap, Cambodia; Centre for Tropical Medicine and Global Health, University of Oxford, Oxford, United Kingdom; Médecins Sans Frontières, New Delhi, India; Department of Infectious Diseases, Christian Medical College, Vellore, India; Nuffield Department of Primary Care Health Sciences, University of Oxford, Oxford, United Kingdom; Department of Community and Family Medicine, All India Institute of Medical Sciences, Patna, India; Department of Infectious Diseases, Christian Medical College, Vellore, India; Nuffield Department of Primary Care Health Sciences, University of Oxford, Oxford, United Kingdom; Centre for Tropical Medicine and Global Health, University of Oxford, Oxford, United Kingdom; Mahidol-Oxford Tropical Medicine Research Unit, Mahidol University, Bangkok, Thailand; Médecins Sans Frontières, New Delhi, India; Department of Clinical Research, London School of Hygiene and Tropical Medicine, London, United Kingdom

**Keywords:** COVID-19, host biomarker, prognosis

## Abstract

Efficient resource allocation is essential for effective pandemic response. We measured host biomarkers in 420 patients presenting with moderate coronavirus disease 2019 and found that different biomarkers predict distinct clinical outcomes. Interleukin (IL)–1ra, IL-6, IL-10, and IL-8 exhibit dose-response relationships with subsequent disease progression and could potentially be useful for multiple use-cases.

Since December 2019, >500 million cases of coronavirus disease 2019 and 6 million deaths have been reported [1]. Unprecedented demand overwhelmed triage rooms, exhausted oxygen supplies, and led to rationing of intensive care [2, 3]. At each level of the health system, optimal resource allocation was paramount.

Biochemical biomarkers of the host response to infection, including those reflecting endothelial activation, immunoregulation, and coagulation, have been shown to be prognostic in a variety of febrile illnesses [4–6] and proposed as risk stratification tools (alone or as adjuncts to clinical risk scores) to help health workers identify patients with a poor prognosis and guide resource allocation [7, 8].

In a previous analysis, we developed and validated 3 readily implementable clinical prediction models for supplemental oxygen requirement in patients presenting with moderate COVID-19 [9]. The models combined 3 simple clinical predictors with a host biomarker for which commercial point-of-care tests are available. In this article, we take a more exploratory approach to evaluate the prognostic utility of 13 biochemical biomarkers, previously found to reflect final common pathways to severe febrile illness and sepsis [10], for predicting a range of disease severities in patients presenting with moderate COVID-19. We hypothesized that different biomarkers would predict distinct clinical outcomes, making them better suited to particular clinical use-cases [11].

## METHODS

### Participant Enrollment

Between 22 October 2020 and 3 July 2021, consecutive adults (aged ≥18 years) presenting to the All India Institute of Medial Sciences (Patna, India) and Christian Medical College Hospital (Vellore, India) with clinically suspected COVID-19 of moderate severity (peripheral oxygen saturation [SpO_2_] ≥94% and respiratory rate [RR] <30 breaths per minute in the context of systemic symptoms [breathlessness or fever and chest pain, abdominal pain, diarrhea, or severe myalgia]) [12, 13], were recruited. Patients who had a prior history of laboratory-confirmed severe acute respiratory syndrome coronavirus 2 (SARS-CoV-2) infection and/or had received at least 1 dose of any COVID-19 vaccine were excluded.

### Data Collection and Follow-up

At enrollment, clinical parameters were measured and venous blood specimens collected in ethylenediaminetetraacetic acid (EDTA) tubes. For participants admitted at the study sites, follow-up was conducted in-person on each day of admission until day 14. For those discharged prior to day 14, follow-up was conducted via telephone on days 7 and 14. Discharged participants reporting worsening symptoms on day 7 and/or persistent symptoms on day 14 were recalled to have their SpO_2_ and RR measured.

### Primary Outcome

The primary endpoint was an ordinal outcome closely aligned with the World Health Organization COVID-19 Clinical Progression Scale (WHO-CPS) [13], reflecting the maximum level of respiratory support a participant required in the 14 days following enrollment. Outcome categories were defined as (1) no supplemental oxygen required (SpO_2_ ≥94% *and* RR <30 *and* SpO_2_/fraction of inspired oxygen [FiO_2_] ≥400; WHO-CPS ≤4); (2) supplemental oxygen required (SpO_2_ <94% *or* RR ≥30 *or* SpO_2_/FiO_2_ <400; WHO-CPS = 5); (3) noninvasive ventilation (NIV; WHO-CPS = 6); and (4) mechanical ventilation (MV) and/or death (WHO-CPS ≥7).

### Laboratory Procedures

Venous blood samples were used to measure complete blood counts on site (XP-300-Hematology-Analyzer, Sysmex). Within 4 hours of collection, the remaining sample was centrifuged and EDTA-plasma aliquots stored at ≤ −20°C. Frozen plasma aliquots were transported on dry ice and concentrations of host biomarkers were quantified (SimplePlex Ella microfluidic platform, ProteinSimple; suPARnostic enzyme-linked immunosorbent assay, Virogates).

Host biomarkers were selected for analysis following review of the literature, expert consultation, and in collaboration with FIND, the global alliance for diagnostics (Geneva, Switzerland). Biomarkers that demonstrated promising prognostic utility in COVID-19 were selected, including those reflecting endothelial activation (angiopoietin-2 [Ang-2]) [14]; immunoregulation (CXCL-10 [IP-10], interleukin 1 receptor antagonist [IL-1ra], interleukin 6 [IL-6], interleukin 8 [IL-8], interleukin 10 [IL-10], soluble triggering receptor expressed on myeloid cells 1 [sTREM-1], soluble urokinase plasminogen activator receptor [suPAR]) [15–19]; inflammation (C-reactive protein [CRP], neutrophil-to-lymphocyte ratio [NLR], procalcitonin [PCT]) [20, 21]; and coagulation (D-dimer, platelets) [20].

### Statistical Analyses

A formal sample size calculation was not required for this secondary exploratory analysis. All analyses were prespecified in the published statistical analysis plan (doi: 10.17605/OSF.IO/DXQ43). Univariable logistic regression was used to quantify the ability of each biomarker to discriminate (c-statistic with 95% confidence interval) participants who developed increasingly severe pulmonary dysfunction, in accordance with the primary outcome categories. Analyses were performed in R version 4.1.2 software.

## RESULTS

Among 2808 adults with clinically suspected SARS-CoV-2 infection, the eligibility rate was 15.9% (446/2808), with 426 participants enrolled (refusal rate 4.5% [20/446]). Three participants who were lost to follow-up (3/426 [0.7%]) and 3 participants with significant comorbidities or coinfections (2 active malignancies with neutropenia and 1 acute pyelonephritis; 3/423 [0.7%]) were excluded, leaving 420 participants for analysis (Supplementary Figure 1). All participants had laboratory-confirmed SARS-CoV-2 infection.

Median age was 53 years (interquartile range [IQR], 41–62 years), and 68% (285/420) of the cohort was male. Two-thirds of participants reported preexisting comorbidities (279/420 [66%]). Median duration of symptoms prior to enrollment was 6 days (IQR 4–8 days), and 93.8% (414/420) had a quick Sequential Organ Failure Assessment score ≤1 at recruitment; no participants had hypoxia or tachypnea, and all were breathing room air (Table 1).

**Table 1.** Baseline Clinical and Laboratory Characteristics of Participants With Moderate Coronavirus Disease 2019 Stratified by Outcome Category

Characteristic	Overall (N = 420)	Outcome Category			
		1	2	3	4
		No Supplemental O_2_ Requirement (n = 331)	Supplemental O_2_ Requirement (n = 62)	Noninvasive Ventilation (n = 15)	Mechanical Ventilation and/or Death (n = 12)
Age, y	53.0 (41.0–62.0)	53.0 (40.5–61.5)	53.5 (42.0–64.8)	51.0 (45.0–65.5)	59.5 (38.8–64.0)
BMI^a^, kg/m²	25.4 (23.5–28.3)	25.4 (23.6–28.6)	25.3 (22.3–28.0)	26.8 (24.0–28.2)	26.5 (24.4–27.3)
Male sex, No. (%)	285/420 (68%)	218/331 (66%)	48/62 (77%)	13/15 (87%)	6/12 (50%)
Heart rate, beats/min	86.0 (78.0–95.0)	86.0 (78.0–94.0)	90.0 (84.0–97.8)	82.0 (78.0–89.0)	87.0 (73.0–102.5)
Respiratory rate, breaths/min	22.0 (22.0–24.0)	22.0 (22.0–24.0)	24.0 (22.0–24.0)	22.0 (22.0–24.0)	23.0 (22.0–24.0)
Oxygen saturation, %	98.0 (96.0–99.0)	98.0 (97.0–99.0)	96.0 (95.0–97.0)	97.0 (96.0–98.0)	96.0 (96.0–98.2)
Systolic BP, mm Hg	126.0 (115.0–134.0)	126.0 (115.5–135.0)	123.0 (115.2–132.8)	130.0 (117.0–140.0)	125.0 (116.5–130.8)
Axillary temperature, °C	36.9 (36.6–37.1)	36.8 (36.5–37.1)	36.9 (36.5–37.2)	37.0 (36.8–37.1)	37.1 (36.9–37.8)
qSOFA score ≥2, No. (%)	26/420 (6.2%)	17/331 (5.1%)	7/62 (11%)	1/15 (6.7%)	1/12 (8.3%)
Duration of illness, d	6.0 (4.0–8.0)	6.0 (4.0–8.0)	6.0 (4.2–7.0)	5.0 (4.5–7.5)	5.0 (4.0–5.0)
Comorbidity, No. (%)	279/420 (66%)	216/331 (65%)	43/62 (69%)	10/15 (67%)	10/12 (83%)
Platelet count^a^, × 10^9^ cells/L	199.0 (147.0–261.0)	207.5 (156.0–268.2)	175.0 (126.0–246.5)	159.0 (126.5–184.5)	170.0 (88.8–187.0)
White cell count^a^, × 10^9^ cells/L	6.2 (4.6–7.8)	6.2 (4.7–7.6)	6.6 (4.6–9.8)	5.0 (3.4–5.9)	6.8 (3.9–9.2)
Neutrophil count^a^, × 10^9^ cells/L	4.0 (2.8–5.7)	4.0 (2.8–5.4)	4.9 (3.2–7.5)	3.7 (2.6–4.3)	5.8 (2.9–6.4)
Lymphocyte count^a^, × 10^9^ cells/L	1.3 (0.9–1.9)	1.4 (1.0–2.0)	1.1 (0.7–1.4)	0.9 (0.6–1.0)	1.1 (0.6–1.4)
NLR^a^	3.1 (1.8–5.1)	2.7 (1.7–4.4)	4.7 (3.2–7.4)	4.6 (3.0–5.8)	4.0 (2.2–9.0)
Ang-2^a^, pg/mL	1688.0 (1237.0–2306.8)	1688.0 (1237.0–2225.0)	1645.0 (1266.8–2529.5)	1367.0 (1166.5–2003.0)	3095.0 (2004.5–6996.5)
CRP^a^, mg/L	37.7 (6.8–107.8)	26.0 (5.5–87.8)	83.0 (27.7–158.0)	49.7 (23.2–130.2)	79.1 (69.5–159.4)
CXCL-10^a^, pg/mL	977.5 (377.5–1951.2)	766.5 (311.5–1532.5)	1955.5 (989.0–2661.5)	2641.0 (1763.0–3445.5)	2566.0 (2009.5–3188.0)
D-dimer^a^, ng/mL	847.3 (467.0–1520.2)	735.7 (410.1–1358.8)	1115.1 (788.4–2334.3)	970.1 (751.3–1674.2)	2612.6 (1153.5–4802.7)
IL-1ra^a^, pg/mL	1000.5 (591.0–1838.0)	841.5 (543.2–1535.0)	1688.5 (998.8–2588.8)	2433.0 (1087.0–3405.5)	3879.0 (2016.0–6333.0)
IL-6^a^, pg/mL	19.5 (6.5–47.0)	13.6 (5.2–36.5)	43.0 (19.3–81.6)	68.9 (29.6–73.2)	89.6 (56.2–148.0)
IL-8^a^, pg/mL	10.6 (7.8–15.6)	9.8 (7.4–13.9)	13.9 (9.2–18.3)	14.3 (9.6–22.1)	25.1 (20.2–31.6)
IL-10^a^, pg/mL	8.4 (5.6–15.1)	7.1 (5.2–12.5)	13.8 (9.4–20.2)	17.2 (11.2–22.0)	28.1 (22.1–29.4)
PCT^a^, pg/mL	103.5 (70.1–164.0)	98.7 (68.2–149.8)	122.0 (80.5–188.2)	133.0 (88.3–295.0)	319.0 (180.0–961.0)
sTREM-1^a^, pg/mL	390.0 (271.0–562.2)	376.0 (262.8–536.0)	437.0 (316.5–649.5)	380.0 (245.5–470.0)	586.0 (395.0–1000.5)
suPAR, ng/mL	4.2 (3.1–5.7)	3.9 (2.9–5.3)	5.7 (3.9–6.7)	5.0 (4.5–5.6)	6.1 (3.8–10.0)
Seronegative^a,b^, No. (%)	188/409 (46%)	139/323 (43%)	27/59 (46%)	12/15 (80%)	10/12 (83%)

Data are presented as median (interquartile range) for continuous variables.

Abbreviations: Ang-2, angiopoietin-2; BMI, body mass index; BP, blood pressure; CRP, C-reactive protein; IL, interleukin; NLR, neutrophil-to-lymphocyte ratio; O_2_, oxygen; PCT, procalcitonin; qSOFA, quick Sequential Organ Failure Assessment; sTREM-1, soluble triggering receptor expressed on myeloid cells 1; suPAR, soluble urokinase plasminogen activator receptor.

a Missing data: BMI = 1 (category 1); platelet count, white cell count, neutrophil count, lymphocyte count, NLR = 10 each (category 1 = 7; category 2 = 3); Ang-2, CXCL-10, IL-1ra, IL-6, IL-8, IL-10, PCT, sTREM-1 = 2 each (category 1 = 1; category 4 = 1); D-dimer = 3 (category 1 = 2, category 4 = 1); CRP = 8 (category 1); serostatus = 11 (category 1 = 8, category 2 = 3).

b Seronegative defined as negative for both immunoglobulin G and immunoglobulin M antibodies against severe acute respiratory syndrome coronavirus 2 (SCoV-2 Detect enzyme-linked immunosorbent assay, InBios).

Most participants did not progress to require supplemental oxygen (category 1; 331/420 [78.8%]). Of 89 participants whose clinical condition deteriorated, 62 required supplemental oxygen (category 2; 62/420 [14.8%]), another 15 received NIV (category 3; 15/420 [3.6%]), and a further 2 were mechanically ventilated and 10 died (category 4; 12/420 [2.9%]).

**Figure 1.**
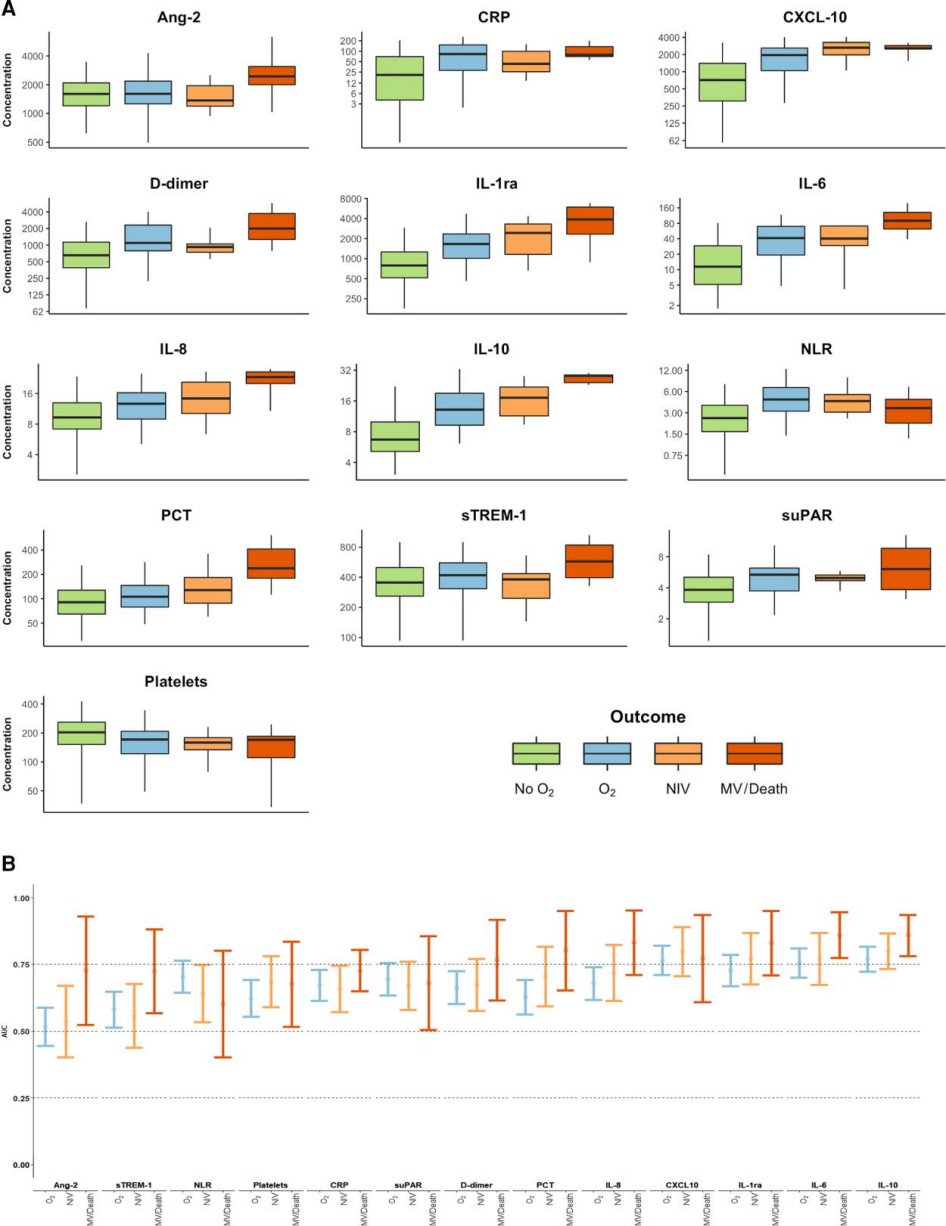
Baseline concentration and prognostic utility of host biomarkers in participants with moderate coronavirus disease 2019. *A*, Concentrations plotted on log_2_ scale and expressed in pg/mL (Ang-2, CXCL-10, IL-1ra, IL-6, IL-8, IL-10, PCT, sTREM-1), ng/mL (D-dimer, suPAR), mg/L (CRP), or × 10^9^ cells/L (platelets). *B*, Biomarkers ordered from left to right by ascending mean c-statistic across the 3 severity outcomes. Abbreviations: Ang-2, angiopoietin-2; AUC, area under the receiver operating characteristic curve; CRP, C-reactive protein; IL, interleukin; MV, mechanical ventilation; NIV, noninvasive ventilation; NLR, neutrophil-to-lymphocyte ratio; O_2_, oxygen; PCT, procalcitonin; sTREM-1, soluble triggering receptor expressed on myeloid cells 1; suPAR, soluble urokinase plasminogen activator receptor.

For all biomarkers, baseline concentrations were different across outcome categories (Table 1), with the majority exhibiting a trend toward higher baseline concentrations in participants who progressed to more severe disease (Figure 1*A*). An inverse trend was observed in platelet count. Some biomarkers (eg, Ang-2, PCT, sTREM-1) demonstrated baseline concentrations that were only notably elevated in participants who developed the most severe clinical phenotypes (category 4), whereas for others (eg, CXCL-10, suPAR, CRP), baseline concentrations appeared to increase substantially between participants in outcome categories 1 and 2, and further increases in participants who progressed to categories 3 (NIV) or 4 (MV and/or death) were less apparent.

The prognostic potential of the biomarkers is summarized in Figure 1*B* and Supplementary Table 2. Consistent with Figure 1*A*, biomarkers that demonstrated dose-response relationships between their baseline concentrations and the maximum level of subsequent respiratory support appeared to have best discrimination (eg, IL-1ra, IL-6, IL-10). The prognostic performance of many of the biomarkers varied depending on the severity of disease predicted.

## DISCUSSION

We report the prognostic utility of 13 biochemical biomarkers in patients presenting with moderate COVID-19 to 2 hospitals in India. Our results indicate that different biomarkers might predict different disease severities and may be suited to distinct clinical use-cases. Biomarkers that discriminate between patients who progress to require supplemental oxygen (CXCL-10, suPAR, CRP, platelet count) may be most helpful to support community-based triage (tests that can rule out need for hospitalization with low negative likelihood ratios), whereas those that predict more severe illness (NIV, MV, or death; PCT, D-dimer, sTREM-1, Ang-2) may be better deployed to guide inpatient resource allocation (ruling-in need for frequent monitoring and/or admission to restricted-capacity high-dependency care areas with high positive likelihood ratios). Biomarkers with dose-response relationships with subsequent disease progression demonstrated promising discrimination across a range of disease severities (IL-10, IL-6, IL-1ra, IL-8) and are particularly attractive candidates for further exploration as they could potentially address multiple use-cases.

The apparent stepwise reduction in NLR in patients who progressed to more severe illness is difficult to reconcile. It may be a function of the relatively small number of events and higher proportion (n = 10 [2.4%]) of missing data for NLR. Nevertheless, if confirmed, it suggests that in the presence of a low NLR, a second parameter (clinical or biomarker) would be required to identify patients at risk of disease progression.

We measured biomarker concentrations at the time of presentation, when no patient had a supplemental oxygen requirement, allowing us to confidently evaluate the prognostic potential of the biomarkers [22]. Clinically, all patients had moderate severity disease at the time of blood collection, suggesting that rather than being crude biochemical surrogates for bedside assessment, certain biomarkers might reflect subclinical pathophysiological changes and may add value to clinical risk scores.

In our context, the only available treatment known to influence the natural course of SARS-CoV-2 infection was corticosteroids [23]. However, steroid use was not associated with disease progression and is unlikely to have confounded the results (Supplementary Table 3). Previous studies have illustrated longitudinal changes in biomarker concentrations during a COVID-19 illness [18]. It is possible that the prognostic performance of the biomarkers may vary accordingly. However, we did not find differences in biomarker concentrations between patients who presented in the first versus second week of their illness (Supplementary Table 4). Although we censored follow-up after day 14, no further disease progression occurred beyond this point. We combined MV and death into a single outcome category due to few surviving ventilated patients. This loss of granularity may have underestimated biomarker discrimination [24]. Combining MV and death was preferable to combining NIV and MV; NIV may have had a less consistent threshold for initiation as it could be provided outside the intensive care unit setting. Vaccinated and previously infected individuals, as well as those with more recent variants, were not included; hence, caution is required if findings are extrapolated to these populations. It is likely that similar pathophysiological pathways (and biomarkers) are implicated, although this requires empirical testing and disease progression will be more frequent in unvaccinated and immune-naive cohorts.

Our results indicate that a number of host biomarkers implicated in the pathophysiology of other acute febrile illnesses may also play a role in the natural history of SARS-CoV-2 infections. The prognostic utility of a particular biomarker is not a standalone concept and is inextricably linked to the clinical outcome(s) being predicted. Further studies should build on our results and investigate the value that biomarker measurements may add to clinical risk scores for well-defined clinical use-cases.

## Supplementary Material

ofac526_Supplementary_Data

## Supplementary Data

Supplementary materials are available at *Open Forum Infectious Diseases* online. Consisting of data provided by the authors to benefit the reader, the posted materials are not copyedited and are the sole responsibility of the authors, so questions or comments should be addressed to the corresponding author.

## Notes

***Author contributions.*** A. C., R. M., L. M., C. K., Y. L., and S. B. conceptualized the study. A. C., L. M., C. K., and S. B. wrote and approved the statistical analysis plan. A. C. and L. M. did the formal analysis. A. C. and A. B. prepared the figures. A. C., L. M., C. K., and S. B. verified the underlying data. A. C. wrote the original draft of the manuscript. All authors reviewed, edited, and approved the manuscript.

***Acknowledgments.*** We are grateful to the members of the External Advisory Panel, led by Prof. Nicholas J. White (Mahidol-Oxford Tropical Medicine Research Uni [MORU]), for their advice and guidance throughout the study.

***Patient consent.*** All patients provided informed written consent for their participation. This investigator-initiated study was prospectively registered (ClinicalTrials.gov: NCT04441372), with protocol and statistical analysis plan uploaded to the Open Science Framework platform (doi: 10.17605/OSF.IO/DXQ43). Ethical approval was given by the All India Institute of Medical Sciences, Patna Ethics Committee; Christian Medical College Ethics Committee; Oxford Tropical Research Ethics Committee; and Médecins Sans Frontières (MSF) Ethical Review Board.

***Data sharing.*** De-identified, individual participant data from this study will be available to researchers whose proposed purpose of use is approved by the data access committees at MSF and MORU. Inquiries or requests for the data may be sent to data.sharing@london.msf.org and datasharing@tropmedres.ac. Researchers interested in accessing biobanked samples should contact the corresponding authors, who will coordinate with the respective institutions.

***Financial support.*** The PRIORITISE (Prognostication of Oxygen Requirement in Patients with Non-severe SARS-CoV-2 Infection) study was funded by Médecins Sans Frontières India, which maintained a sponsor/investigator role for the study. The Wellcome Trust provides core funding to MORU in Bangkok (220211, 215604/Z/19/Z), which supported the design, monitoring, and analysis of the study. C. K. is supported by a Wellcome Trust/Royal Society Sir Henry Dale Fellowship (211182/Z/18/Z). For the purpose of open access, the author has applied a CC BY public copyright license to any author-accepted manuscript version arising from this submission.
