# Correction to: Host Biomarkers Reflect Prognosis in Patients Presenting With Moderate Coronavirus Disease 2019: A Prospective Cohort Study

**DOI:** 10.1093/ofid/ofad037

**Published:** 2023-04-18

In the original publication of this article, [Chandna A, Mahajan R, Gautam P et al. Host Biomarkers Reflect Prognosis in Patients Presenting With Moderate Coronavirus Disease 2019: A Prospective Cohort Study, Open Forum Infectious Diseases, Volume 9, Issue 10, October 2022, ofac526, https://doi.org/10.1093/ofid/ofac526], the members of the PRIORITISE study investigative group were not listed as contributors under their group in the author byline. The members of this group have been added in the version of the article currently available online, and the publisher regrets this error.

