# Supplementary material for: Host Biomarkers Reflect Prognosis in Patients Presenting With Moderate Coronavirus Disease 2019: A Prospective Cohort Study
Source: Open Forum Infect Dis. 2022 Oct 6;9(10):ofac526. doi: 10.1093/ofid/ofac526 (PMC9605705; doi:10.1093/ofid/ofac526)
Supplement: ofac526_Supplementary_Data [file ofac526_supplementary_data.docx]

**Host biomarkers reflect prognosis in patients presenting with moderate covid-19 – a prospective cohort study**

**SUPPLEMENTARY APPENDIX**

**CONTENTS PAGE**

1. **PRIORITISE study investigators** 2
2. **Study recruitment flow chart** 4
3. **Discrimination of host biomarkers for different clinical outcomes** 5
4. **Association of corticosteroid use with increasing levels of respiratory support** 6
5. **Baseline concentrations of host biomarkers stratified by week of illness** 7
6. **STROBE checklist** 9

**Supplementary Table 1. PRIORITISE study investigators.**

| **PRIORITISE Study Investigators** | |
| --- | --- |
| **Name** | **Primary Affiliation** |
| Kundavaram PP Abhilash | Department of Emergency Medicine, Christian Medical College, Vellore, India |
| T Balamugesh | Department of Pulmonary Medicine, Christian Medical College, Vellore, India |
| Sakib Burza | Médecins Sans Frontières, New Delhi, India |
| Atanu Bhattacharjee | Mahidol-Oxford Tropical Medicine Research Unit, Mahidol University, Bangkok, Thailand |
| Divendu Bhusan | Department of Internal Medicine, All India Institute of Medical Sciences, Patna, India |
| Arjun Chandna | Cambodia Oxford Medical Research Unit, Angkor Hospital for Children, Siem Reap, Cambodia |
| Arthur T L Cheung | Mahidol-Oxford Tropical Medicine Research Unit, Mahidol University, Bangkok, Thailand |
| Thambu David | Department of Medicine, Christian Medical College, Vellore, India |
| Nicholas Day | Mahidol-Oxford Tropical Medicine Research Unit, Mahidol University, Bangkok, Thailand |
| Divya Dayanand | Department of Infectious Diseases, Christian Medical College, Vellore, India |
| Sabine Dittrich | Foundation for Innovative Diagnostics, Geneva, Switzerland |
| Arjen Dondorp | Mahidol-Oxford Tropical Medicine Research Unit, Mahidol University, Bangkok, Thailand |
| Priyanka Gautam | Department of Infectious Diseases, Christian Medical College, Vellore, India |
| Tulasi Geevar | Department of Transfusion Medicine & Immunohaematology, Christian Medical College, Vellore, India |
| Biju George | Department of Haematology, Christian Medical College, Vellore, India |
| Srinivasa R Ghattamaneni | Médecins Sans Frontières, New Delhi, India |
| Karthik Gunasekaran | Department of Medicine, Christian Medical College, Vellore, India |
| Richa Gupta | Department of Respiratory Medicine, Christian Medical College, Vellore, India |
| Samuel G Hansdak | Department of Medicine, Christian Medical College, Vellore, India |
| Samreen Hussain | Médecins Sans Frontières, New Delhi, India |
| Ramya Iyadurai | Department of Medicine, Christian Medical College, Vellore, India |
| Carolina Jimenez | Médecins Sans Frontières, New Delhi, India |
| Rajiv Karthik | Department of Infectious Diseases, Christian Medical College, Vellore, India |
| Rohini Karthikeyan | Department of Infectious Diseases, Christian Medical College, Vellore, India |
| Sharwar Kazmi | Médecins Sans Frontières, New Delhi, India |
| Constantinos Koshiaris | Nuffield Department of Primary Care Health Sciences, University of Oxford, Oxford, UK |
| Pragya Kumar | Department of Community & Family Medicine, All India Institute of Medical Sciences, Patna, India |
| Sanjeev Kumar | Department of Cardiothoracic & Vascular Surgery, All India Institute of Medical Sciences, Patna, India |
| Shiril Kumar | Department of Virology, Rajendra Memorial Research Institute of Medical Sciences, Patna, India |
| Vikash Kumar | Médecins Sans Frontières, New Delhi, India |
| Debasree Kundu | Department of Infectious Diseases, Christian Medical College, Vellore, India |
| Ankita Lakshmanan | Médecins Sans Frontières, New Delhi, India |
| Yoel Lubell | Mahidol-Oxford Tropical Medicine Research Unit, Mahidol University, Bangkok, Thailand |
| Raman Mahajan | Médecins Sans Frontières, New Delhi, India |
| Abi Manesh | Department of Infectious Diseases, Christian Medical College, Vellore, India |
| Chonticha Menggred | Mahidol-Oxford Tropical Medicine Research Unit, Mahidol University, Bangkok, Thailand |
| Mahesh Moorthy | Department of Clinical Virology, Christian Medical College, Vellore, India |
| Lazaro Mwandigha | Nuffield Department of Primary Care Health Sciences, University of Oxford, Oxford, UK |
| Jennifer Osborn | Foundation for Innovative Diagnostics, Geneva, Switzerland |
| Melissa Richard-Greenblatt | Perelman School of Medicine, University of Pennsylvania, Philadelphia, USA |
| Sowmya Sathyendra | Department of Medicine, Christian Medical College, Vellore, India |
| Merylin Sebastian | Department of Infectious Diseases, Christian Medical College, Vellore, India |
| Sadhana Sharma | Department of Biochemistry, All India Institute of Medical Sciences, Patna, India |
| Veena K Singh | Department of Burns & Plastic Surgery, All India Institute of Medical Sciences, Patna, India |
| Vikash K Singh | Médecins Sans Frontières, New Delhi, India |
| Christopher Smith | School of Tropical Medicine & Global Health, Nagasaki University, Nagasaki, Japan |
| Javvad Suri | Médecins Sans Frontières, New Delhi, India |
| Shuichi Suzuki | School of Tropical Medicine & Global Health, Nagasaki University, Nagasaki, Japan |
| Jaruwan Tubprasert | Mahidol-Oxford Tropical Medicine Research Unit, Mahidol University, Bangkok, Thailand |
| Paul Turner | Cambodia Oxford Medical Research Unit, Angkor Hospital for Children, Siem Reap, Cambodia |
| George Varghese | Department of Infectious Diseases, Christian Medical College, Vellore, India |
| Annavi M G Villanueva | School of Tropical Medicine & Global Health, Nagasaki University, Nagasaki, Japan |
| Naomi Waithira | Mahidol-Oxford Tropical Medicine Research Unit, Mahidol University, Bangkok, Thailand |
| Anand Zachariah | Department of Medicine, Christian Medical College, Vellore, India |

**Supplementary Figure 1. Screening and enrolment of participants.** *Other reasons for exclusion: 3 = unable to provide informed consent; 5 = reason not documented.

**
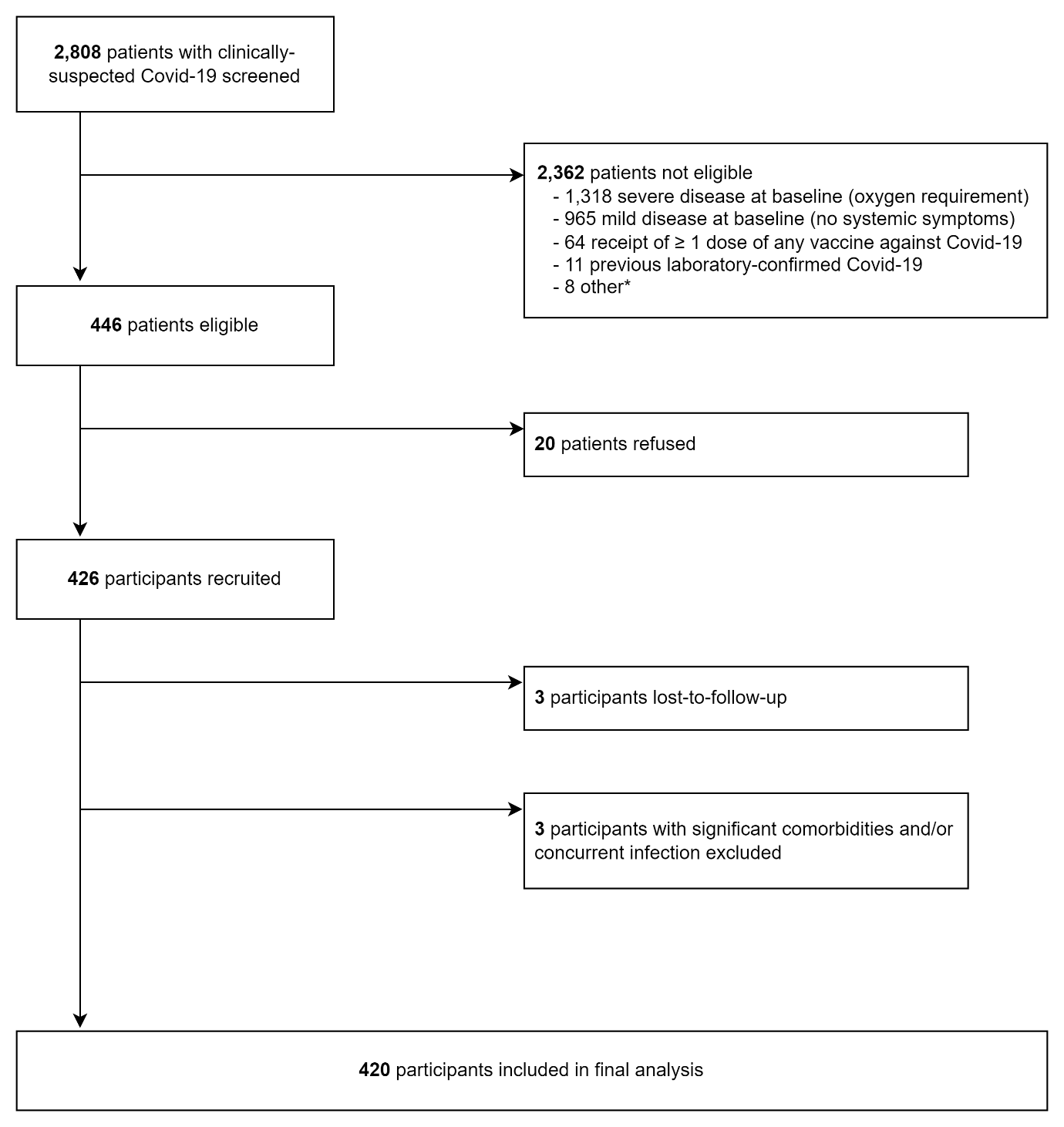
**

**Supplementary Table 2. Discrimination of host biomarkers for different clinical outcomes.** ^*^Missing data (in baseline group unless otherwise stated): Platelet count, NLR = 10 (Supplemental O_2_ Requirement = 3/10); Ang-2, CXCL-10, IL-1ra, IL-6, IL-8, IL-10, PCT, sTREM-1 = 2 (MV and/or Death = 1/2); D-dimer = 3 (MV and/or Death = 1/3); CRP = 8. AUC = area under the receiver operating characteristic curve; CI = confidence interval; MV = mechanical ventilation; N = number of events; NIV = non-invasive ventilation.

| **Biomarker** | **AUC** (95% CI) | | |
| --- | --- | --- | --- |
|  | **Supplemental O_2_ Requirement**  N = 89 | **Non-invasive ventilation**  N = 27 | **Mechanical ventilation and/or Death**  N = 12 |
| **Ang-2** ^*^  (pg/ml) | 0.52  (0.44 - 0.59) | 0.54  (0.40 - 0.67) | 0.73  (0.52 - 0.93) |
| **CRP** ^*^  (mg/l) | 0.67  (0.61 - 0.73) | 0.66  (0.57 - 0.74) | 0.73  (0.65 - 0.80) |
| **CXCL10** ^*^  (pg/ml) | 0.77  (0.71 - 0.82) | 0.80  (0.71 - 0.89) | 0.77  (0.61 - 0.94) |
| **D-dimer** ^*^  (ng/ml) | 0.66  (0.60 - 0.72) | 0.67  (0.58 - 0.77) | 0.77  (0.62 - 0.92) |
| **IL-1ra** ^*^  (pg/ml) | 0.73  (0.67 - 0.79) | 0.77  (0.68 - 0.87) | 0.83  (0.71 - 0.95) |
| **IL-6** ^*^  (pg/ml) | 0.75  (0.70 - 0.81) | 0.77  (0.67 - 0.87) | 0.86  (0.77 - 0.95) |
| **IL-8** ^*^  (pg/ml) | 0.68  (0.62 - 0.74) | 0.72  (0.61 - 0.82) | 0.83  (0.71 - 0.95) |
| **IL-10** ^*^  (pg/ml) | 0.77  (0.72 - 0.82) | 0.80  (0.73 - 0.87) | 0.86  (0.78 - 0.94) |
| **NLR** ^*^ | 0.70  (0.64 - 0.76) | 0.64  (0.53 - 0.75) | 0.60  (0.40 - 0.80) |
| **Platelet count** ^*^  (x10^9^ cells/l) | 0.62  (0.55 - 0.69) | 0.68  (0.59 - 0.78) | 0.68  (0.52 - 0.84) |
| **PCT** ^*^  (pg/ml) | 0.63  (0.56 - 0.69) | 0.70  (0.59 - 0.82) | 0.80  (0.65 - 0.95) |
| **sTREM-1** ^*^  (pg/ml) | 0.58  (0.51 - 0.65) | 0.56  (0.44 - 0.68) | 0.72  (0.57 - 0.88) |
| **suPAR**  (ng/ml) | 0.69  (0.63 - 0.75) | 0.67  (0.58 - 0.76) | 0.68  (0.51 - 0.86) |

**Supplementary Table 3. Association of corticosteroid use with increasing levels of respiratory support.** Corticosteroid use is defined as use of oral or parenteral steroids, occurring at least one calendar day prior to developing an oxygen requirement (for participants that received oxygen therapy). ^*^Chi-squared test. CI = confidence interval.

|  | | **Met outcome** | | | **Relative risk**  **(95% CI)** | **p-value** ^*^ |
| --- | --- | --- | --- | --- | --- | --- |
|  |  | Yes | No | **TOTAL** |  |  |
| **Supplemental oxygen requirement** | | | | | | |
| **Corticosteroid use** | Yes | 32 | 125 | **157** | 0.94  (0.64 - 1.38) | 0.75 |
|  | No | 57 | 206 | **263** |  |  |
|  | **TOTAL** | **89** | **331** | **420** |  |  |
| **Non-invasive ventilation** | | | | | | |
| **Corticosteroid use** | Yes | 9 | 148 | **157** | 0.84  (0.39 - 1.82) | 0.65 |
|  | No | 18 | 245 | **263** |  |  |
|  | **TOTAL** | **27** | **393** | **420** |  |  |
| **Mechanical ventilation and/or death** | | | | | | |
| **Corticosteroid use** | Yes | 4 | 153 | **157** | 0.84  (0.26 - 2.74) | 0.77 |
|  | No | 8 | 255 | **263** |  |  |
|  | **TOTAL** | **12** | **408** | **420** |  |  |

**Supplementary Table 4. Baseline concentrations of host biomarkers stratified by week of illness.** Median (IQR) reported for continuous variables. ^*^Missing data: Platelet count, White cell count, Neutrophil count, Lymphocyte count, NLR = 10 (Week 1 = 4; Week 2 = 6); Ang-2, CXCL-10, IL-1ra, IL-6, IL-8, IL-10, PCT, sTREM-1 = 2 (Week1 = 1; Week 2 = 1); D-dimer = 3 (Week 1 = 2, Week 2 = 1); CRP = 8 (Week 1); serostatus = 11 (Week 1 = 7, Week 2 = 4). ^†^Seronegative defined as negative for both IgG and IgM antibodies against SARS-CoV-2.

| **Characteristic** | **Overall**  N = 420 | **Days of Symptoms Prior to Presentation** | |
| --- | --- | --- | --- |
|  |  | **≤ 7 days**  N = 299 | **> 7 days**  N = 121 |
| **Platelet count** ^*^  (x10^9^ cells/l) | 199.0  (147.0, 261.0) | 190.0  (143.0, 246.0) | 230.0  (166.0, 309.0) |
| **White cell count** ^*^  (x10^9^ cells/l) | 6.2  (4.6, 7.8) | 5.9  (4.5, 7.3) | 6.8  (5.2, 9.2) |
| **Neutrophil count** ^*^  (x10^9^ cells/l) | 4.0  (2.8, 5.7) | 3.7  (2.8, 5.3) | 4.8  (3.4, 6.7) |
| **Lymphocyte count** ^*^  (x10^9^ cells/l) | 1.3  (0.9, 1.9) | 1.3  (0.9, 1.9) | 1.4  (1.0, 1.9) |
| **NLR** ^*^ | 3.1  (1.8, 5.1) | 2.9  (1.7, 4.7) | 3.3  (2.2, 5.9) |
| **Ang-2** ^*^  (pg/ml) | 1,688.0  (1,237.0, 2,306.8) | 1,603.5  (1,212.5, 2,195.5) | 1,927.5  (1,325.8, 2,493.5) |
| **CRP** ^*^  (mg/l) | 37.7  (6.8, 107.8) | 36.4  (6.6, 96.0) | 39.8  (9.3, 133.4) |
| **CXCL10** ^*^  (pg/ml) | 977.5  (377.5, 1,951.2) | 1,034.5  (418.8, 2,120.0) | 766.5  (310.5, 1,558.0) |
| **D-dimer** ^*^  (ng/ml) | 847.3  (467.0, 1,520.2) | 847.3  (457.5, 1,484.5) | 861.5  (476.8, 1,598.1) |
| **IL-1** ^*^  (pg/ml) | 1,000.5  (591.0, 1,838.0) | 1,094.0  (625.5, 1,991.2) | 826.0  (503.2, 1,487.8) |
| **IL-6** ^*^  (pg/ml) | 19.5  (6.5, 47.0) | 20.3  (6.9, 51.2) | 16.7  (5.2, 43.4) |
| **IL-8** ^*^  (pg/ml) | 10.6  (7.8, 15.6) | 10.5  (7.9, 16.0) | 10.6  (7.4, 13.9) |
| **IL-10** ^*^  (pg/ml) | 8.4  (5.6, 15.1) | 9.1  (5.7, 16.1) | 7.4  (5.3, 11.9) |
| **PCT** ^*^  (pg/ml) | 103.5  (70.1, 164.0) | 106.0  (70.2, 168.5) | 98.6  (70.0, 147.0) |
| **sTREM-1** ^*^  (pg/ml) | 390.0  (271.0, 562.2) | 377.5  (259.2, 530.5) | 441.5  (306.2, 633.2) |
| **suPAR**  (ng/ml) | 4.2  (3.1, 5.7) | 3.9  (2.9, 5.5) | 4.6  (3.4, 5.9) |
| **Seronegative** ^*†^ | 46%  (188 / 409) | 50%  (147 / 292) | 35%  (41 / 117) |

**STROBE Statement—Checklist of items that should be included in reports of *cohort studies***

|  | Item No | Recommendation | Page No |
| --- | --- | --- | --- |
| **Title and abstract** | 1 | (*a*) Indicate the study’s design with a commonly used term in the title or the abstract | 1, 3 |
|  |  | (*b*) Provide in the abstract an informative and balanced summary of what was done and what was found |  |
| Introduction | | | |
| Background/rationale | 2 | Explain the scientific background and rationale for the investigation being reported | 4 |
| Objectives | 3 | State specific objectives, including any prespecified hypotheses | 4 |
| Methods | | | |
| Study design | 4 | Present key elements of study design early in the paper | 5 |
| Setting | 5 | Describe the setting, locations, and relevant dates, including periods of recruitment, exposure, follow-up, and data collection | 5, Fig S1 |
| Participants | 6 | (*a*) Give the eligibility criteria, and the sources and methods of selection of participants. Describe methods of follow-up | 5 |
|  |  | (*b*) For matched studies, give matching criteria and number of exposed and unexposed |  |
| Variables | 7 | Clearly define all outcomes, exposures, predictors, potential confounders, and effect modifiers. Give diagnostic criteria, if applicable | 5, 6 |
| Data sources/ measurement | 8* | For each variable of interest, give sources of data and details of methods of assessment (measurement). Describe comparability of assessment methods if there is more than one group | 5, 6 |
| Bias | 9 | Describe any efforts to address potential sources of bias | 5, 6 |
| Study size | 10 | Explain how the study size was arrived at | 7 |
| Quantitative variables | 11 | Explain how quantitative variables were handled in the analyses. If applicable, describe which groupings were chosen and why | 7 |
| Statistical methods | 12 | (*a*) Describe all statistical methods, including those used to control for confounding | 7 |
|  |  | (*b*) Describe any methods used to examine subgroups and interactions |  |
|  |  | (*c*) Explain how missing data were addressed |  |
|  |  | (*d*) If applicable, explain how loss to follow-up was addressed |  |
|  |  | (*e*) Describe any sensitivity analyses |  |
| Results | | |  |
| Participants | 13* | (a) Report numbers of individuals at each stage of study—eg numbers potentially eligible, examined for eligibility, confirmed eligible, included in the study, completing follow-up, and analysed | 8, Fig S1 |
|  |  | (b) Give reasons for non-participation at each stage |  |
|  |  | (c) Consider use of a flow diagram |  |
| Descriptive data | 14* | (a) Give characteristics of study participants (eg demographic, clinical, social) and information on exposures and potential confounders | 8, Table S2 |
|  |  | (b) Indicate number of participants with missing data for each variable of interest |  |
|  |  | (c) Summarise follow-up time (eg, average and total amount) |  |
| Outcome data | 15* | Report numbers of outcome events or summary measures over time | 8 |

| Main results | 16 | (*a*) Give unadjusted estimates and, if applicable, confounder-adjusted estimates and their precision (eg, 95% confidence interval). Make clear which confounders were adjusted for and why they were included | 8, 9  Fig 1,  Table S3 |
| --- | --- | --- | --- |
|  |  | (*b*) Report category boundaries when continuous variables were categorized |  |
|  |  | (*c*) If relevant, consider translating estimates of relative risk into absolute risk for a meaningful time period |  |
| Other analyses | 17 | Report other analyses done—eg analyses of subgroups and interactions, and sensitivity analyses | Table S4 |
| Discussion | | | |
| Key results | 18 | Summarise key results with reference to study objectives | 10 |
| Limitations | 19 | Discuss limitations of the study, taking into account sources of potential bias or imprecision. Discuss both direction and magnitude of any potential bias | 10, 11 |
| Interpretation | 20 | Give a cautious overall interpretation of results considering objectives, limitations, multiplicity of analyses, results from similar studies, and other relevant evidence | 11 |
| Generalisability | 21 | Discuss the generalisability (external validity) of the study results | 11 |
| Other information | | | |
| Funding | 22 | Give the source of funding and the role of the funders for the present study and, if applicable, for the original study on which the present article is based | 12, 13 |

*Give information separately for exposed and unexposed groups.

**Note:** An Explanation and Elaboration article discusses each checklist item and gives methodological background and published examples of transparent reporting. The STROBE checklist is best used in conjunction with this article (freely available on the Web sites of PLoS Medicine at http://www.plosmedicine.org/, Annals of Internal Medicine at http://www.annals.org/, and Epidemiology at http://www.epidem.com/). Information on the STROBE Initiative is available at <http://www.strobe-statement.org>.
